# Aborted Cardiac Arrest in LQT2 Related to Novel *KCNH2* (*hERG*) Variant Identified in One Lithuanian Family

**DOI:** 10.3390/medicina57070721

**Published:** 2021-07-16

**Authors:** Neringa Bileišienė, Jūratė Barysienė, Violeta Mikštienė, Eglė Preikšaitienė, Germanas Marinskis, Monika Keževičiūtė, Algirdas Utkus, Audrius Aidietis

**Affiliations:** 1 Clinic of Cardiac and Vascular Diseases, Institute of Clinical Medicine, Faculty of Medicine, Vilnius University, Santariškių str. 2, LT-08661 Vilnius, Lithuania; jurate.barysiene@santa.lt (J.B.); germanas.marinskis@santa.lt (G.M.); audrius.aidietis@santa.lt (A.A.); 2 Department of Human and Medical Genetics, Institute of Biomedical Sciences, Faculty of Medicine, Vilnius University, Santariskiu g. 2, LT-08661 Vilnius, Lithuania; violeta.mikstiene@santa.lt (V.M.); egle.preiksaitiene@santa.lt (E.P.); algirdas.utkus@santa.lt (A.U.); 3 Center of Cardiology and Angiology, Vilnius University Hospital Santaros Klinikos, Santariskiu g. 2, LT-08661 Vilnius, Lithuania; monika.kezeviciute@santa.lt

**Keywords:** long QT syndrome type 2, *KCNH2*, sudden cardiac death, mutation

## Abstract

Congenital long QT syndrome (LQTS) is a hereditary ion channelopathy associated with ventricular arrhythmia and sudden cardiac death starting from young age due to prolonged cardiac repolarization, which is represented by QT interval changes in electrocardiogram (ECG). Mutations in human ether-à-go-go related gene (*KCNH2* (7q36.1), formerly named *hERG*) are responsible for Long QT syndrome type 2 (LQT2). LQT2 is the second most common type of LQTS. A resuscitated 31-year-old male with the diagnosis of LQT2 and his family are described. Sequencing analysis of their genomic DNA was performed. Amino acid alteration p.(Ser631Pro) in *KCNH2* gene was found. This variant had not been previously described in literature, and it was found in three nuclear family members with different clinical course of the disease. Better understanding of genetic alterations and genotype-phenotype correlations aids in risk stratification and more effective management of these patients, especially when employing a trigger-specific approach to risk-assessment and individually tailored therapy.

## 1. Introduction

Cardiac channelopathies exist in young patients without apparent structural heart disease who are usually unaware of their disorder, and sudden cardiac (SCD) death might be the first presenting symptom for them [1]. The most common cardiac channelopathy is LQTS, which can be either congenital or acquired. Congenital LQTS is considered to be rare, with the prevalence of 1 in 2500 in the general population [2]. However, the frequency might be underestimated due to the incomplete penetrance of genetic alleles. Genetic testing has become essential part of diagnosis for hereditary cardiac arrhythmia syndromes.

Congenital LQTS (ORPHA:768) is a clinical disorder of genetic origin characterized by delayed repolarisation of the myocardium, electrocardiographic QT prolongation, and increased risk of syncope, seizures, and SCD caused by polymorphic ventricular tachycardia, known as Torsades des Pointes (TdP) [3]. To date, pathogenic variants associated with LQTS have been identified in 19 genes responsible for LQTS: 15 following an autosomal-dominant pattern of inheritance (*AKAP9* (MIM# 604001), *ANK2* (MIM# 604001), *CACNA1C* (MIM# 604001), *CALM1* (MIM# 604001), *CALM2* (MIM# 604001), *CALM3* (MIM# 604001), *CAV3* (MIM# 604001), *KNCE2* (MIM# 604001), *KCNH2* (MIM# 604001), *KCNJ2* (MIM# 604001), *KCNJ5* (MIM# 604001), *RYR2* (MIM# 604001), *SCN1B* (MIM# 604001), *SCN4B* (MIM# 604001), *SCN5A* (MIM# 604001), and *SNTA1* (MIM# 604001)), one following an autosomal-recessive pattern (TRDN), and two following both autosomal-dominant and-recessive patterns (*KCNQ1* and *KCNE1*) [4,5]. LQT1, LQT2, and LQT3 genotypes comprise more than 95% of the patients with genotype-positive LQTS and approximately 75% of all patients with LQTS [6]. Most patients with LQTS can be successfully treated with beta-blockers, life-style interventions (e.g., avoidance of QT-prolonging drugs, hypokalaemia and hypomagnesemia, limiting physical activity) and implantable cardioverter defibrillators (ICDs) for cases with high-risk clinical features [7].

LQT2, the second most common type of LQTS (up to 30%), is caused by heterozygous mutation in the *KCNH2* (*hERG*) gene (MIM#152427) on chromosome 7q36 [2,8]. *KCNH2* (*hERG*) gene encodes the pore-forming α-subunit of the rapidly activating delayed rectifier cardiac potassium current IKr channels, which is a major determinant of cardiac action potential duration in the mammalian heart. The majority of alterations in *KCNH2* (*hERG*) increase the transmural dispersion of repolarization and eventually induce prolongation of the QT interval, a notched T wave in ECG, re-entry tachyarrhythmia, and TdP [5]. Mutations in the *KCNH2* pore-loop region, which is responsible for channel’s ion conduction pathway, carry extremely high risk of threatful cardiac events. *KCNH2* gene missense variant, in which amino acid serine at position 631 (pore-loop region) is substituted with alanine (Ser-631-Ala), is reported to be linked to a long QT syndrome [9]. In this case report, we present alternative missense substitution in the *KCNH2* gene NM_000238.3:c.1891T > C, NP_000229.1:p.(Ser631Pro), rs199472959 identified in three nuclear family members with different clinical presentations.

## 2. Materials and Methods

Ethical approval and informed consent. This study has been approved by The Vilnius Regional Biomedical Research Ethics Committee and The Ethics Committee of Vilnius University Hospital Santaros Clinics (VUH SK). Proband and his mother gave their written informed consent for inclusion; assent and permission for inclusion was obtained from the daughter’s parents.

Genetic counselling. In 2019, the proband was referred to a clinical geneticist with the clinical diagnosis of LQTS. Genetic testing results revealed likely pathogenic variant of the *KCNH2* gene; thus, investigation was assigned to the proband’s mother and his daughter.

Clinical evaluation consisted of previous medical and family history, standard 12-lead ECGs, transthoracic echocardiography, exercise tolerance test, and a 24-h Holter monitoring. Diagnoses were established in accordance with the current ESC Guidelines for the management of patients with ventricular arrhythmias and the prevention of sudden cardiac death [10]. QTc intervals were estimated according to Bazett’s formula, calculating the heart rate-corrected QT interval, which remains preferable for diagnosis and prognosis in LQT1 and 2 patients [11].

Next-generation sequencing. Next-generation sequencing analysis of genomic DNA isolated from the proband’s peripheral blood was performed using TruSight Cardio Se-quecing panel (Illumina Inc., San Diego, CA, USA). A total of 174 genes (coding exons) were analysed, including the main genes associated with long QT. Prepared DNA libraries were sequenced on Illumina MiSeq system 9 (Illumina Inc., San Diego, CA, USA). Data analysis was performed using standard Illumina bioinformatic workflow (Illumina Inc., San Diego, CA, USA). Detected gene variants were analysed and annotated using VariantStudio 3.0 software (Illumina Inc., San Diego, CA, USA). Synonymous or intronic variants and variants with a minor allele frequency >2% have been excluded. In silico analysis of missense variants was performed using PolyPhen-2 (http://genetics.bwh.harvard.edu/pph2/ (accessed on 12 October 2020), SIFT Human Protein (http://sift.jcvi.org/ (accessed on 12 October 2020) and Mutation Taster (www.mutationtaster.org/ (accessed on 12 October 2020) tools.

Sanger sequencing. Polymerase chain reaction (PCR) of gDNA sequences flanking *KCNH2* gene variant NM_000238.3:c.1891T > C, NP_000229.1:p.(Ser631Pro), rs199472959 was performed using specific primers designed with Primer Blast tool [12]. The PCR products were sequenced using BigDye^®^ Terminator v3.1 Cycle Sequencing Kit (Thermo Fisher Scientific, Waltham, MA, USA) and ABI 3130xL Genetic Analyser (Thermo Fisher Scientific, Waltham, MA, USA). The sequences were aligned with the reference sequence of the *KCNH2* (NCBI: NM_000238.3) gene.

Review of the literature. PubMed database was searched for articles, using keywords “LQT2 phenotype genotype”. Papers mainly from the past five years were reviewed, and articles relevant to presented case were analysed; the reference list was composed using Mendeley.

## 3. Case Report

A 31-year-old Caucasian male was transferred to VUH SK intensive cardiac care unit from a non-PCI centre after successful resuscitation and an intravenous fibrinolysis for suspected acute coronary syndrome (ACS). From the patient’s anamnesis, it was known that he suddenly collapsed at his workplace, where he was working as manager, in the morning hours. According to the Emergency Medical Service (EMS) documents, the patient was unconscious, hemodynamically unstable, and defibrillated. On admission to VUH SK, laboratorial tests revealed slightly elevated Troponin I values (818 ng/L normal range ≤ 35 ng/L), normal value of brain natriuretic peptide (15 ng/L, normal range ≤ 100ng/L in acute heart failure), and persisting prominent hypokalaemia (in non-PCI centre 3.03 mmol/L, normal range 3.5–5.5 mmol/L; in VUH SK 3.4 mmol/L; normal range 3.8–5.3 mmol/L). The 12-lead ECG on admission to VUH SK showed sinus rhythm with a rate of 104 beats per minute and significant QTc prolongation (532 ms) while applying therapeutic hypothermia. To rule out ACS diagnosis, coronary angiography was performed. No abnormalities were found in coronary arteries. Transthoracic echocardiography was consistent with structurally normal heart with normal ejection fraction.

Patient’s anamnesis revealed convulsive syncope six months prior to this event. During this episode, the patient lost consciousness while smoking and talking with his colleagues at work during morning hours and recovered spontaneously. The patient was referred to a neurologist in an out-patient clinic and encephalography was performed, but the diagnosis of epilepsy was not confirmed. From family history, the patient’s brother experienced a number of convulsive syncopes although he had never consulted with doctors. At the age of 43, the patient’s brother died suddenly.

During the hospitalization, QT prolongation persisted on the patient’s repeated 12-lead ECGs even after stopping of therapeutic hypothermia and correction of electrolyte imbalance. The diagnosis of long QT syndrome was established (Scoring System for Clinical Diagnosis of LQTS—5 points). In addition to the treatment with beta-blockers, an ICD was implanted. Bearing in mind the patient’s family history, genetic counselling was assigned. Next-generation sequencing identified a heterozygous NM_000238.4:c.1891T > C, NP_000229.1:p.(Ser631Pro) and likely pathogenic variant of the *KCNH2* gene. The biochemical difference between serine and proline is moderate (Grantham score 74). In silico analysis results: SIFT—pathogenic (score 0), PolyPhen2—likely pathogenic (score 0.617), Mutation Taster—pathogenic (score 0.99). The frequency of the variant in the NHLBI Exome Sequencing Project group, 1000 Genomes Project is 0.0; GnomAD database frequency 0.

**Figure 1:**
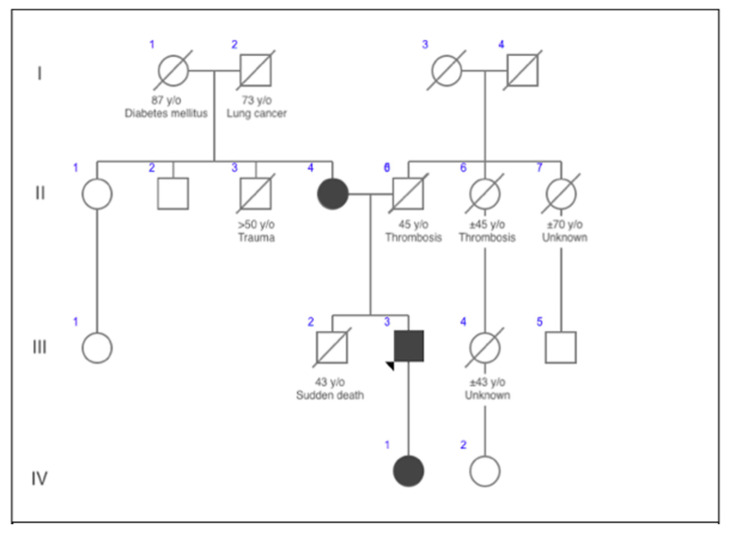
Pedigree of the case. The arrow marks the proband. In the presented case, the proband (III3), his mother (II4), and his daughter (IV1) all carried the same novel heterozygous NM_000238.3:c.1891T > C, NP_000229.1:p.(Ser631Pro), likely pathogenic variant of the KCNH2 gene. Sudden cardiac death of proband’s brother (III2) aged 43 is known. Relevant information regarding age and death causes of relatives obtained from the family history is marked above particular individual.

**Figure 2:**
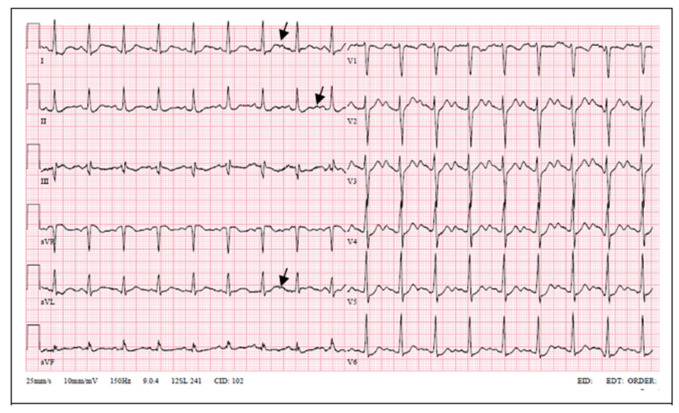
Patient’s ECG on admission (notched T waves, QTc 532 ms).

**Figure 3:**
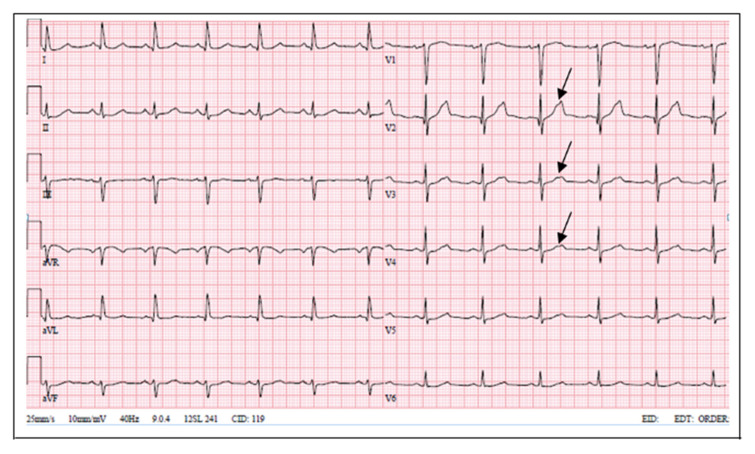
Patient’s mother ECG (notched T waves, QTc 489 ms).

**Figure 4:**
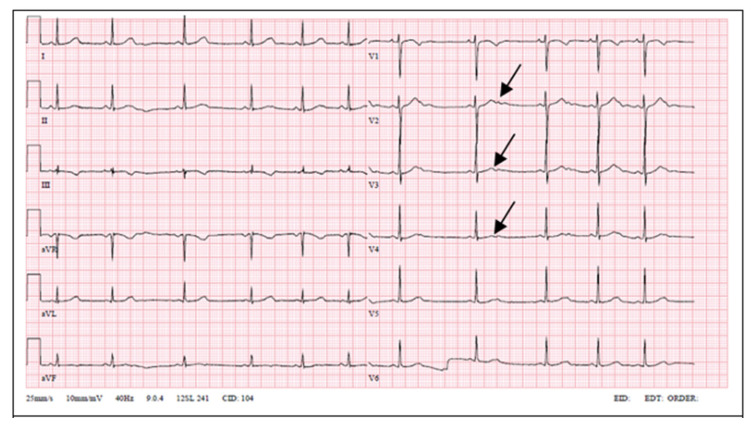
Patient’s daughter ECG (low-amplitude notched T waves, QTc 423 ms).

Considering this variant to be responsible for the clinical presentation to the proband, a segregation analysis for family members was recommended. Sanger sequencing identified the same heterozygous variant for the patient’s 67-year-old mother and 10-year-old daughter. Pattern of inheritance of the mutation through several generations is provided in the form of a family tree in Figure 1. Their ECGs are provided in Figure 2, Figure 3 and Figure 4. Medical history of the patient’s mother revealed that she underwent recurrent syncopes (six or more episodes, typically in the mornings) during post-partum period at the ages between 23–27. She associated these symptoms with experienced domestic violence. After genetic testing, the proband’s mother was referred to a cardiologist, and LQT2 diagnosis was established (Scoring System for Clinical Diagnosis of LQTS—6 points). Long-term treatment with beta-blockers was prescribed. Also she underwent implantation of an ICD. The proband’s daughter remains asymptomatic with no previous history of arrhythmias or loss of consciousness. Currently, she is on therapy with beta-blockers.

## 4. Discussion

According to prof. P. J. Schwartz, an experienced cardiologist should be able to guess with reasonable accuracy the genotype of LQTS on the basis of clinical history, circumstances associated with the cardiac event, and sometimes the T-wave morphology on electrocardiogram, but confirmation must be sought from laboratory testing [13]. In this case, typical changes in QT segment seen in the patient’s ECGs and clinical history enabled us to establish the diagnosis of LQTS with high suspicion of LQT2.

LQT2 carriers show low-amplitude T waves with high incidence of notches in their ECGs [14]. Bifid T waves are the hallmark of the LQT2 genotype. Furthermore, in LQT2 T-wave amplitude is commonly low, and the QT interval ranges from normal to markedly prolonged (QTc 470 ± 30 ms) [15]. Our patient had prolonged QT interval and notched T waves. His mother’s ECG showed similar changes; meanwhile, the proband’s daughter’s ECG showed notched and low-amplitude T waves without QT interval prolongation.

LQT2 patients are at higher risk of arrhythmias when their potassium levels are low [13]. On admission to non-PCI centre, blood serum potassium of the patient was only 3.03 mmol/L. Serum potassium levels should be restored and maintained above 4 mmol/L in patients with LQT2.

*KCNH2* (*hERG*) mutation carriers often experience cardiac events during early morning hours. These events are frequently elicited by loud noises, such as sirens and alarm clocks [5], and are most prevalent in females with LQT1 and LQT2 from all LQTS patients. Despite the fact that overall rate of cardiac events related to LQT1/LQT2 is higher in women, LQT2-related lethal events occur more frequently in men (2% vs. 6%) [16]. In our presented case, the patient’s mother has not experienced aborted cardiac arrest as patient did. 

All women with LQTS have increased risk for arrhythmia during the first nine months post-partum, with LQT2 genotype patients being at the highest risk group [17]. The patient’s mother’s syncopal episodes started in her post-partum period. The risk for recurrent syncope among LQT2 women is also significantly increased during perimenopausal and the early (five years after onset) postmenopausal periods compared with the patients in their reproductive period [18], but proband’s mother could not specify her physical status during this period, and she probably had not experienced major cardiac events during this time.

The patient’s daughter was asymptomatic before treatment with beta-blockers and remains asymptomatic during her pre-puberty. There is an opinion that LQT2 women may be especially sensitive to oestrogen activity, which may result in an increased risk for arrhythmogenic events after the onset of adolescence because oestrogen was shown to exhibit both acute and genomic effects on I_Kr_, including reduction in channel function and prolongation of ventricular repolarization [8].

Genomic effects on phenotype vary in LQT2 patients. During genetic testing, we found missense variant NM_000238.3:c.1891T > C, NP_000229.1:p.(Ser631Pro), rs199472959 located in the *KCNH2* gene region encoding intramembrane pore-forming segment H5 of the protein. This novel variant causes amino acid change in the evolutionarily conserved position of the protein, which might impact its secondary structure due to the differences of these residues in polarity, charge, size, and/or other characteristics. In silico analysis predicts the variant to be probably deleterious to the protein structure and/or function. An alternative missense variant in the same residue (p.(Ser631Ala)) has been reported in the Human Gene Mutation Database linked with a long QT syndrome [19], supporting the functional importance of this residue. Further functional analyses would be warranted to elucidate the particular consequences of the alteration.

According to the nature of the gene alterations, a bit more than half of LQT2-linked mutations are nonsense type, and most of them lead to haploinsufficiency via nonsense-mediated RNA decay, while the remaining LQT2-linked changes are rare missense variants (representing about 90% in LQT2) causing amino acid substitutions, which disrupt the trafficking of the full-length Kv11.1 channel proteins to the cell surface membrane [7].

*KCNH2*-encoded channel Kv11.1 is composed of amino acid residues from 398 through 657 (S5-loop-S6 region: 552–657), with the N-terminus region defined before residue 398 and the C-terminus region after residue 657. According to other authors, the pore region of the *hERG* channel was defined as the area extending from S5 to the mid-portion of S6, involving amino acid residues 550 through 650 [20].

Our identified missense variant in *KCNH2* gene is located in the intramembrane pore region. To the best of our knowledge, this variant has not been previously defined in the scientific literature nor listed in NHLBI Exome Sequencing, 1000G projects, or GnomAD databases. The genetic change was classified as likely pathogenic to the criteria of the American College of Medical Genetics and Genomics (ACMG). Therefore, it is suspected to result in altered inward potassium current, leading to prolongation of repolarization demonstrated in ECG and eventually leading to cardiac arrhythmia and other clinical symptoms. Moss et al. investigated the clinical features and prognostic implications of variants involving the pore and non-pore regions of the *hERG* channel in 201 LQT2 subjects and found that patients with pore mutations had a markedly increased risk for arrhythmia-associated cardiac events (syncope, cardiac arrest, or sudden death) compared to those with non-pore mutations [21]. Furthermore, Shimizu et al. demonstrated in their study of 858 LQT2 patients that the QTc interval was longer, and cardiac events were more frequent in patients with mutations in the transmembrane pore locations (S5-loop-S6) than in those with mutations in either transmembrane non-pore (S1–S4), N-terminus, or C-terminus locations. Moreover, the QTc interval was longer in patients with amino acid substitutions compared to frameshift/nonsense or other genetic alterations. SCD was also more frequent in patients with missense variants [22]. Migdalovich et al. analysed data of 1166 subjects with genetically confirmed *KCNH2* variants and demonstrated that women experienced a high rate of life-threatening events regardless of mutation location (pore-loop: 35%, non-pore loop: 23%), whereas in men, the rate of aborted cardiac arrest or SCD was higher among those with pore-loop mutations (28%). Possible mechanisms that may explain the observed gender-related differences include the fact that oestrogen increases I_Kr_ independently of variant location, thereby increasing arrhythmic risk even among women who carry lower-risk (non-pore) mutations in the *KCNH2* channel. In contrast, the protective effects of testosterone on I_Kr_ and ventricular repolarization in post-adolescent males result in a reduction in the risk of arrhythmic events among carriers of low-risk mutations, with a possible remaining residual risk in men who harbour higher risk mutations in the functionally more important pore-loop region [8]. Nevertheless, according to Moss et al., risk of arrhythmia-associated cardiac events is higher in pore (region) mutations and persists throughout the first 40 years of life, with the risk increasing by increasing QTc duration [21]. Interestingly, missense mutations in LQT2 were not associated with a statistically significant increase in the risk for either arousal or exercise-triggered cardiac events [23], as in the presented case. 

Even with the current knowledge in molecular biology, the treatment of LQT2 patients remain challenging. Beta-blockers’ efficacy in a time-dependent manner within risk- and age-subsets have demonstrated a pronounced 64% reduction in the risk of cardiac events in high-risk patients with the LQT2 genotype [24]. It is generally known that propranolol or nadolol are the preferred beta blockers for therapy of LQTS, particularly for patients with LQT1 or LQT2. To the best of our knowledge, there are not enough data analysing the effects of potassium supplementation or administration of spironolactone or progesterone in LQT2 patients [6].

In the presented case, all three family members received treatment in accordance with current ESC guidelines for the management of patients with ventricular arrhythmias and the prevention of sudden cardiac death [10]. Lifestyle changes were recommended, and beta-blockers were administered for all of them. An implantable cardioverter defibrillator was implanted for the proband, who experienced SCD, and his mother, with known history of previous syncope episodes and documented QTc on ECG > 500 ms, thus considering her to be a carrier of likely pathogenic variant in *KCNH2.* Despite the fact that cardiac events in our patients were not associated with arousal-triggers, we strongly agree that avoidance of unexpected auditory stimuli, such as sudden loud noise, alarm clocks, and phone ringing tones, should be advised in all individuals diagnosed with LQT2, especially in post-adolescent women with alterations in the pore segment of the *hERG* channel [6].

## 5. Conclusions

The novel *KCNH2* (*hERG*) variant p.(Ser631Pro) located in the S6/pore region was identified in a patient diagnosed with LQTS based on typical ECG changes and clinical features.
